# A Data‐Driven Approach to the Issue of ‘Catching Up With Monolinguals’

**DOI:** 10.1111/1460-6984.70330

**Published:** 2026-09-29

**Authors:** Cécile De Cat, Arief Gusnanto, Ludovica Serratrice, Philippe Prévost, Laurie Tuller, Sharon Unsworth

**Affiliations:** ^1^ University of Leeds Leeds West Yorkshire UK; ^2^ University of Oxford Oxford Oxfordshire UK; ^3^ University of Tours Tours, Centre‐Val de Loire France; ^4^ Radboud University Nijmegen Gelderland the Netherlands

**Keywords:** bilingual education, language assessment, language exposure, morphosyntax, vocabulary development

## Abstract

**Background:**

Multilingual children in monolingual education systems are generally assessed against monolingual norms, raising questions about identifying genuine language support needs versus expected developmental trajectories.

**Aims:**

This study aimed to identify: (1) the amount of cumulative exposure to the societal language (SL) beyond which multilingual children perform within the range of monolinguals (at group level) in terms of SL proficiency, and (2) the point at which cumulative SL experience no longer correlates with SL proficiency outcomes.

**Methods:**

We examined multilingual learners (ages 5–9 years) across three societal languages (English, French, Dutch), assessing morphosyntax via sentence repetition tasks and receptive vocabulary measures (breadth and depth). Binary partition and broken stick regression models were employed to identify thresholds in the exposure‐outcome relationship while controlling for environmental and cognitive factors affecting both monolingual and multilingual children. Cumulative SL exposure was measured in month‐equivalents.

**Results:**

For morphosyntax, at least 59 month‐equivalents of cumulative SL exposure was required for multilingual children's mean performance to fall within the range of monolingual variation in SL proficiency. Diminishing returns occurred after approximately 57 months (years). For vocabulary, no clear or reliable thresholds emerged. Outcomes were more strongly predicted by cognitive factors and richness of language experience than cumulative exposure alone.

**Conclusions:**

Continued SL support throughout primary education is recommended, with full group‐level comparability to monolingual peers in morphosyntax suggested beyond approximately 93 month‐equivalents. These findings provide research‐informed guidance for practitioners to contextualise language assessment expectations based on multilingual children's language backgrounds, moving away from deficit‐based comparisons.

**WHAT THIS PAPER ADDS:**

*What is already known on this subject*

- Previous research has revealed considerable variation in the amount of time needed for multilingual children in monolingual education settings to ‘catch up’ with their monolingual peers in terms of school language proficiency. However, that research is based on arbitrary cut‐offs, and the extent to which it accounts for other factors (besides the amount of exposure to the societal language (SL)) is limited.

*What this study adds to the existing knowledge*

- The innovative use of statistical modelling allowed us to approach this question in a way that avoids deficit‐framing of multilingualism. Informed by a highly diverse cohort of 5‐ to 9‐year‐olds, our study accounts for the variability of performance among monolinguals *as well as* multilinguals, measured with a range of SL proficiency measures, and taking into account a range of environmental and developmental predictors. This revealed that it takes at least 57 month‐equivalents of cumulative SL exposure before multilinguals start performing within the monolingual range for morphosyntactic production.

*What are the clinical implications of this study?*

- These findings provide research‐informed guidance for practitioners to contextualize language assessment expectations based on multilingual children's language backgrounds, recognizing expected developmental trajectories.

## 1 Introduction

The context we are considering in this study is one in which children are growing up with more than one language, while attending a monolingual education system in the societal language. Children growing up in such circumstances are sometimes labelled *heritage bilinguals* (in reference to the language(s) they acquire at home) or, in countries like the UK, children with *English as an additional language* (EAL), and increasingly more often referred to as multilingual learners, the term we adopt in this study. Our starting point is a practical question, faced by speech & language therapists and educators when assessing the language support needs of multilingual children in this context, namely: do some of the children in their care need extra support with the societal language, that is the language of schooling, or might they be at risk for developmental language disorder? (We do not consider support in the children's other language(s), although we acknowledge this is important, and the heritage language is supported by the richness of their language and literacy experiences (e.g., Correia et al. 2025; Torregrossa et al. 2023)). Multilingual learners in linguistically diverse classrooms tend to be highly heterogeneous in terms of the amount of exposure they experience in the SL and the range of language rich opportunities they experience outside of the classroom. They vary in the extent to which the SL becomes their dominant language over time. Because their linguistic experience is distributed across two or more languages, multilingual learners are inevitably going to differ in some way from monolingual learners, who receive 100% of their input in one single language.^1^

Whether comparing multilinguals to monolinguals is justifiable, or even meaningful, has been the subject of much recent debate (see, e.g., Rothman et al. 2023). Western educational systems have long been shaped by a monolingual bias in which the developmental trajectories of monolingual children have been treated as the normative benchmark. As a result, the language practices and competencies of multilingual learners have often been viewed through a deficit lens (e.g., Genesee 2022). In research contexts, Leivada et al. (2023) objected to the systematic comparison of bilinguals to matched monolingual controls, as this can only be done on a small number of dimensions and risks ignoring important ones. They argue that it is essential to acknowledge the diversity among monolinguals, and advocate the use of comparative data across a continuum of bilingualism, taking its multidimensional nature into account through individual and contextual factors. According to these authors, the definition of monolingualism is problematical in the first place, if it does not take into account even small amounts of use of another language, and the changes in language practices across the lifespan.

Our position is not about conforming to theoretically defined norms or comparing multilinguals to an idealised monolingual standard. We embrace diversity across multilingual and monolingual children, as variation is an inherent feature of learning and development. We adopt a working definition of monolingualism as *children whose daily language interactions in the home are exclusively in the societal language*. In this study we focus on officially monolingual societies where—with few exceptions—one societal language is used as the language of schooling in mainstream state education (i.e., English in England, Dutch in the Netherlands, French in France). In these societal contexts there are no special provisions or allowances for multilinguals when it comes to the assessment of language skills or subject specific knowledge. Regardless of language background, the expectations are the same for multilingual and monolingual pupils: the educational system has an intrinsic monolingual bias as expectations are standardised on a population of children who only speak and use one language. Given the current state of affairs, it is not within our remit to change how children are assessed at school. What we can do instead is provide research‐informed evidence on how the diverse and heterogeneous language and literacy experiences of multilingual children predict different outcomes in the language of schooling.

At the heart of our study, there is also an acknowledgment of the variability of monolingual performance. Even for children who have 100% of their input in one single language, environmental factors such as socio‐economic status (e.g., Fernald et al. 2013; Pace et al. 2017; Romeo et al. 2018; Rowe 2008) and child‐level skills like working memory (e.g., Adams and Gathercole 1995; Mooney et al. 2021; Newbury et al. 2016) predict variability in language outcomes.

Finally, there are also factors that are specific to the lived experiences of multilingual learners. While chronological age can be taken as a rough proxy for developmental stages for monolinguals who receive all their input from birth in the societal language, the amount of exposure of multilinguals will vary as a function of age of first exposure to the language and how exposure is distributed across languages over time. Therefore, the cumulative exposure, and not chronological age, will better predict how much linguistic knowledge multilingual children will have acquired at any given point in time (Unsworth 2013). And it is likely that different amounts of cumulative exposure will be necessary for different domains of language. While there is a finite set of morphosyntactic features children need to master a language in terms of grammatical acceptability, it is harder to define an upper bound for the breadth and depth of their lexical knowledge, as these continue increasing through the lifetime. From a pedagogical point of view, a deeper understanding of the relationship between language exposure and language skills would allow practitioners to contextualise expectations for the achievement of bilingual children based on their language background. The question of how long it might take for multilingual learners to ‘catch up’ with their monolingual peers is a question that has received attention in the literature (e.g., Binks and Thomas 2024; Demie 2013; Dixon et al. 2022; Paradis and Jia 2017; Paradis et al. 2017) and it is one that we address in the context of research‐informed expectations in the practical context of a multilingual classroom.

In particular, we aim to identify the amount of experience in the SL beyond which bilingual children can, on average, be expected to perform within the range of monolingual peers in the SL (see, e.g., Thordardottir 2011 for other work along these lines). We focus on morphosyntax (as assessed by a sentence repetition task) and on receptive measures of vocabulary breadth and depth. While these only paint a partial picture of the language knowledge that children need to become competent speakers and access the school curriculum, they are well known measures that underpin more complex linguistic knowledge such as inferential skills (e.g., Valentini and Serratrice 2021).

We investigate the issue of ‘multilinguals catching up with monolinguals’ from two complementary data‐driven perspectives, aiming to identify population‐level benchmarks that contextualise clinical expectations. Each perspective operationalises the notion of ‘catching up’ in a different way. The first perspective frames the question in terms of whether group‐level differences in SL proficiency persist between multilinguals and monolinguals at a given level of cumulative exposure. The second perspective frames the question developmentally. While cumulative SL exposure is a primary driver of early SL acquisition, its predictive strength is not expected to remain constant over time. As children acquire the core structural properties of the SL, the marginal contribution of additional exposure diminishes, and individual differences in outcomes become increasingly attributable to cognitive and environmental factors (the same factors that drive variability among monolinguals). Identifying the point at which this shift occurs is therefore theoretically motivated: it marks the transition from an exposure‐driven developmental phase to one governed by the same factors that shape monolingual trajectories. Accordingly, our research questions are as follows: (1) What is the amount of cumulative exposure to the SL beyond which multilingual children can, on average, be expected to perform within the range of monolingual peers? and (2) From what point does cumulative SL experience no longer correlate with SL proficiency?

## 2 Method

Each research question was addressed in a separate set of analyses described in ‘Modelling methods’ below.

Ethical approval was obtained from the University of Leeds' Faculty of Arts, Humanities & Communications Research Ethics Committee (FAHC 21–006 Amd2); the University of Tours Comité d'Éthique de la Recherche Tours‐Poitiers (No. CER‐TP 2021–09–04); and the Radboud University Ethics Assessment Committee of the Faculty of Arts and the Faculty of Philosophy, Theology and Religious Studies (No. 2021–9263). Informed written consent was obtained from all parents. Children were tested individually at school (or exceptionally in their home) by a research assistant proficient in the respective SL.

### 2.1 Participants

Participants were monolingual (*n* = 95), bilingual (*n* = 93) and trilingual (*n* = 30) children growing up in France, the Netherlands, or the United Kingdom, aged between 5 and 9 years old (mean = 82 months, sd = 12).^2^ Table 1 shows the distribution of mono/bi/trilinguals by societal language. Across the sample, there was a total of 38 heritage languages.^3^ As a proxy for socio‐economic status, we used the highest level of parental education in the household, which was distributed as shown in Table 2.

**TABLE 1:** Number of children included in the analyses.

	Dutch	English	French	Sum
Monolingual	37	39	19	95
Bilingual	41	33	19	93
Trilingual	16	12	2	30
Sum	94	84	40	218

**TABLE 2:** Highest level of parental education in the household (0, one; 1, primary; 2, secondary; 3, post‐secondary; 4, university).

	0	1	2	3	4
France	0	2	11	8	19
Netherlands	1	3	6	10	74
United Kingdom	0	2	12	14	56

Children's exposure to each language was calculated based on information collected via the parental questionnaire Q‐BEx (De Cat et al. 2025). Our main predictor of interest was the cumulative exposure to the SL. This is the number of full‐month‐equivalents of exposure to the SL, obtained as follows: First, the questionnaire respondent defined language periods, based on relevant changes in the child's life. For each period, they were then asked to specify the proportion of exposure of each language. The duration of the period in months was multiplied by that proportion, resulting in full‐month‐equivalents measures for each language. The cumulative exposure to each language was calculated by adding the values obtained for each period.^4^ The distribution of cumulative SL exposure in each country is shown in Table 3. Overall, children had experienced a mean cumulative SL exposure of 44.3 full‐months‐equivalents (SD = 21.8). In the monolingual children (not included in the table), cumulative SL exposure is equivalent to age in months.

**TABLE 3:** Multilingual children's cumulative SL exposure, by country (showing mean and standard deviation).

Country	Mean	SD
France	58.32	17.37
Netherlands	36.95	20.63
United Kingdom	47.07	21.61

### 2.2 Data Exclusions

The original sample included children recruited via speech and language therapy (SLT) clinics in France. These children were excluded from the present study. In the Netherlands, children with a known history of speech and language therapy were excluded from recruitment. In the UK, no such exclusion criterion was applied, and the SLT status of children was unknown. Given these differences in recruitment strategies, it is possible that some of the low performing children in our sample are affected by a language disorder. Q‐BEx calculates a Risk Factor index based on questions about early language development and parental concerns (see De Cat et al. 2026). We flagged children with a Risk Factor index (RF) of ‐3 or lower,^5^ by creating a binary indicator variable. The children at or below RF ‐3 amount to 3 % of the data (7 children). Sensitivity analyses were carried out to estimate the impact of excluding these children (see below).

Parental responses confirmed that all children were currently exposed to the SL of their respective country of residence, that is French, Dutch or English. There were however four children with zero cumulative SL exposure, which we interpreted as missing data for the relevant questions. These children were therefore excluded. Furthermore, 3 children appeared to have unrealistically low cumulative SL exposure.^6^ We excluded these 3 children from the analyses.

### 2.3 SL Proficiency Measures

Morphosyntax outcomes were assessed with the LITMUS sentence repetition test in each societal language (Marinis and Armon‐Lotem 2015). For each child, we report three overall scores: identical repetitions, target repetitions, and grammatical attempts. Identical repetitions require verbatim repetition of the original sentence. Target repetitions require the correct repetition of the target structure of interest in the item (e.g., a relative clause). Grammatical attempts only require the production of a grammatical sentence, even if part of the original sentence was omitted or replaced. These overall scores correspond to the mean across items (*n* = 30 in English and Dutch; *n* = 16 in French).^7^

Vocabulary breadth was measured in the UK with the British Picture Vocabulary Scale (BPVS, Dunn et al. 2009), in France with the Echelle de vocabulaire en images Peabody (EVIP, Dunn et al. 1993), and in the Netherlands with the Peabody Picture Vocabulary Test (PPVT, Dunn et al. 2005).

Vocabulary depth was measured with the Word Classes component of the Clinical Evaluation of Language Fundamentals CELF‐V (in its Dutch, English, or French version—see Kort et al. 2008; Semel et al. 2017; Wiig et al. 2019 respectively).

### 2.4 Control Variables

The control variables included in the regression models were determined by previous analyses reported in Prévost, De Cat, et al. (2026) for sentence repetition outcomes and Serratrice et al. (2026) for the vocabulary outcomes. These analyses used an automated stepwise procedure to identify the best cognitive and environmental predictors of SL outcomes (for each SL proficiency measure in turn). These predictors are used as control variables in the present study. They include information about early language development (whether there was a delay in the production of first words or first sentences and whether the parents had been concerned about the child's language development before age 4), indicators of richness of the SL experience (literacy activities, organised activities, time interacting with friends—see Unsworth et al. 2025), parental education, the presence of older siblings in the home, short‐term and working memory (indexed by forward‐ and backward‐digit repetition tasks—Hill et al. 2021), non‐verbal intelligence (indexed by the Raven matrices—Wechsler 2014, 2013), phonological competence (indexed by the Quasi‐Universal Non‐Word Repetition task from the LITMUS battery—see dos Santos and Ferré 2018), and country of residence. For the specific set of control variables included in each model, please see the Supplementary Materials.

The three countries differ in the age at which formal SL schooling begins. In France, children attend écoles maternelles from the age of three, and primary school (école élémentaire) begins at age six. In the UK, compulsory education begins with the Early Years Foundation Stage in the year children turn five, with primary school starting one year later. In the Netherlands, compulsory education begins at age five, although in practice nearly all children attend primary school from the age of four. These differences in school entry age are one reason why cumulative SL exposure (rather than chronological age) is used as the primary predictor in our models: it captures the actual amount of SL input each child has received regardless of when formal schooling began. To control for between‐country differences, SL was included as a control variable in the models.

### 2.5 Modelling Methods

We used two complementary approaches to analyse the data. The first research question was investigated using a binary partition method, focusing on mean differences between groups, and aiming to ascertain the amount of cumulative SL exposure beyond which that difference in means is no longer significant. For the second research question, we fitted broken stick models, which sought to identify a *change* in the relationship between cumulative SL exposure and proficiency outcomes. We explain each method in detail before turning to the results.

#### 2.5.1 Binary Partition Models

In the first set of analyses, we used a binary partition method to ascertain the amount of cumulative SL exposure beyond which there was no significant difference between monolingual and multilingual children (in the sense that that the cumulative SL exposure variable was no longer a significant predictor of SL proficiency beyond that point). For each proficiency measure in turn, we started from a linear regression model in which cumulative SL exposure was entered (alongside the control variables) as a predictor of SL proficiency. Instead of treating cumulative SL exposure as a continuous variable, we created a binary indicator variable flagging whether cumulative SL exposure exceeded a particular cut‐off point. For example, if the cut‐off point was 80 months of cumulative SL exposure, all the children below that point were treated as ‘bilingual‐like’, while the others were treated as ‘monolingual‐like’. We replaced the cumulative SL exposure variable in the model with this binary indicator variable, and refitted the model recursively, using each time a different threshold. In other words, the cut‐off point separating ‘monolingual‐like’ and ‘bilingual‐like’ children (using the binary indicator variable) were changed each time we fitted the model. As cumulative SL exposure ranged from 6 to 107 month‐equivalents in our population sample, the thresholds we considered ranged from 9 to 94 months of cumulative SL exposure, increasing by 2 months at each iteration (i.e., 9 months, 11, months, 13 months, etc, up to 94 months). The lowest and highest cut‐offs considered (to define the indicator variable) are represented by horizontal lines in Figure 1, which shows the distribution of cumulative SL experience in monolinguals versus multilinguals. The aim of each set of models was to identify the cut‐off point (if any) from which the indicator variable was no longer a significant predictor of SL proficiency.

**FIGURE 1:**
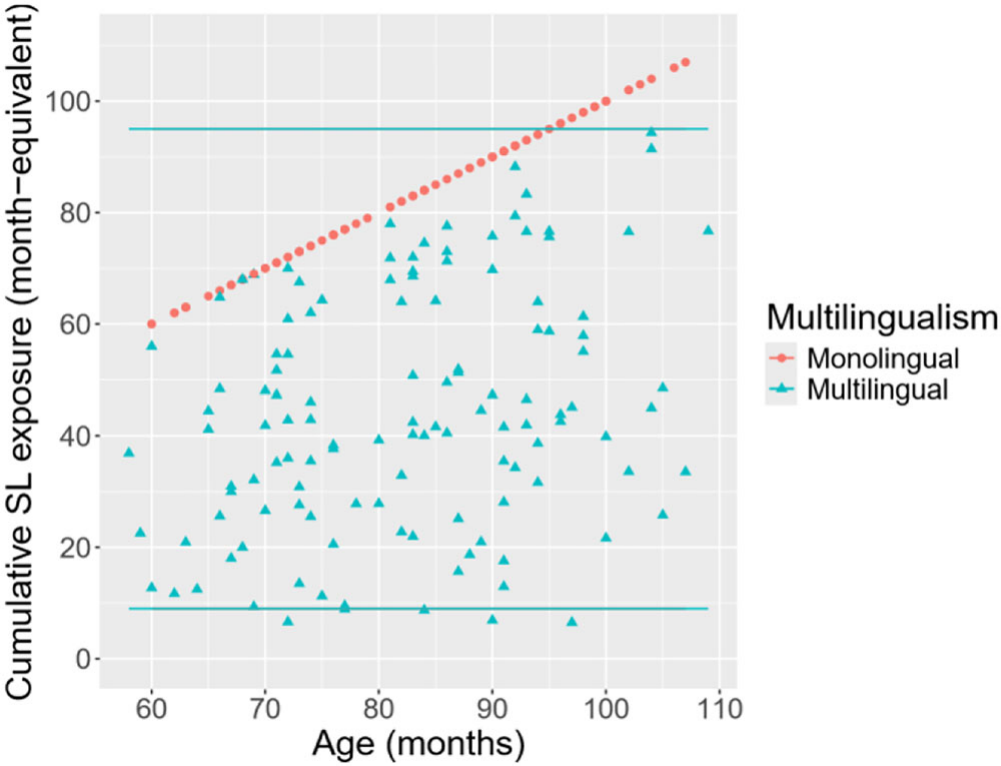
Cumulative SL exposure in monolinguals and multilinguals. The range of cut‐off points considered in the binary partition models is between the two horizontal lines.

#### 2.5.2 Broken Stick Models

In the second set of analyses, we used a broken‐stick approach to bring in a developmental perspective, extrapolated from the cross‐sectional data (Ulm 1991). Our aim was to determine from what point cumulative SL experience no longer correlated with SL proficiency. In broken stick models, the regression line has different slopes before and after the breakpoint. However, the line is continuous because there is only a single point in the line at the breakpoint (i.e., the start of the line after the breakpoint is the same point as the end of the line before the breakpoint, as illustrated in Figure 2).

**FIGURE 2:**
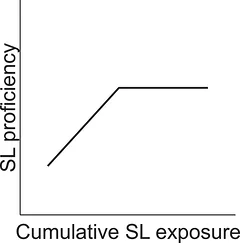
Broken stick illustration.

The breakpoint is not pre‐determined: it is estimated from the data by an iterative algorithm (Muggeo and Muggeo 2008). The breakpoint determined by the model indicates where the relationship between the dependent and independent variables changes. In our case, we sought to determine if there was a particular threshold beyond which cumulative SL exposure was no longer predictive of SL outcomes in the multilingual children. This would be the case if the slope for the second segment was not statistically different from zero (i.e., horizontal, as in Figure 2).

Our first set of analyses (using the binary partition method) is based on different modelling assumptions, as we focus only on significant differences in mean between the two groups (the one below the cut‐off point, and the one above), in terms of SL proficiency outcomes. The broken stick approach, by contrast, assumes that the baseline for the second stick (i.e., slope) starts from where the first stick ends. This prevents the model from allowing a discontinuity (i.e., a hiatus or a ‘jump’) in the relationship between predictor and outcome (which would be akin to non‐continuous development). As our sample did not include monolinguals with less than 60 months of cumulative SL exposure (corresponding to the youngest age in monolingual recruitment), we had to model separate slopes for monolinguals and multilinguals. A single slope would have yielded an artificially low cut‐off point, due to the concentration of monolinguals on the upper‐end of the cumulative SL exposure distribution (as seen in Figure 3).

**FIGURE 3:**
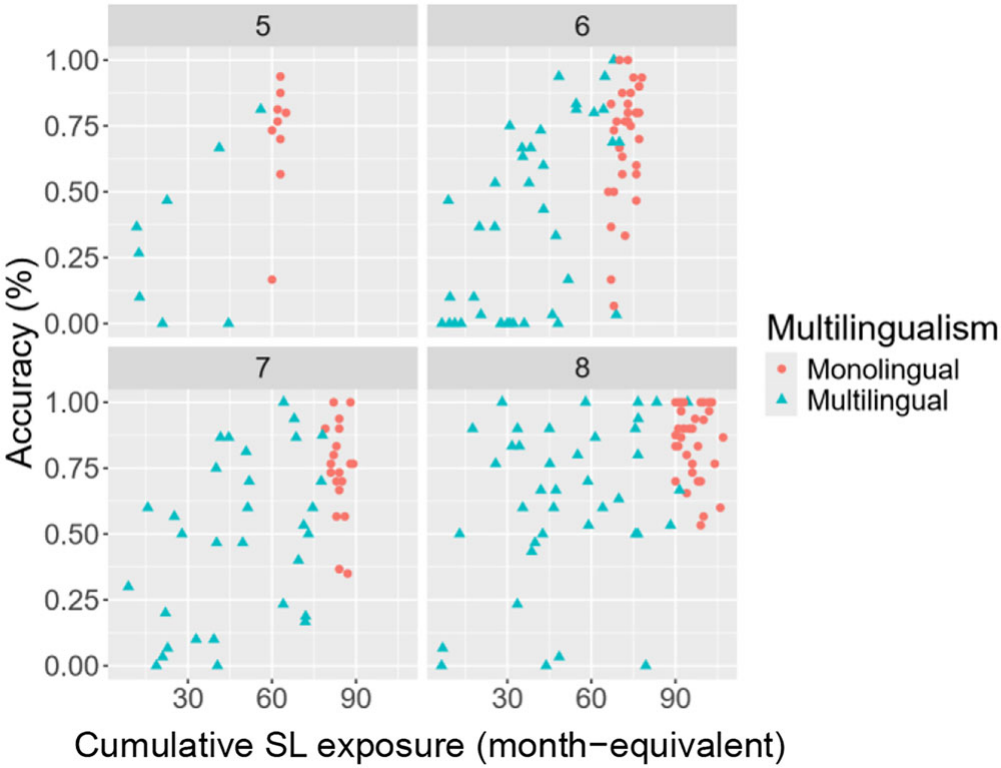
Relationship between accuracy scores for identical repetitions and cumulative SL exposure, by age group (in years) and multilingualism.

All analyses were done in R version 4.4.1 (R Core Team 2024). The binary partition models were fitted with betareg version 3.2.1 (Cribari‐Neto and Zeileis 2010) when the outcome variable was expressed as a proportion, or with lm when the outcome variable was an integer. The broken stick models were fitted with segmented (Fasola et al. 2018; Muggeo and Muggeo 2008), based on lm models (as betareg is currently incompatible with segmented).

## 3 Results

Figure 3 shows the range of performance in the sentence repetition test in monolinguals versus multilinguals, by age group.^8^ Partition by age group is only used here for preliminary visualisation of the data: age is not used as a predictor in any of the analyses below (instead, development is taken into account via language exposure and cognitive development). While cumulative SL exposure equates to age in the monolinguals, it varies widely in all age groups in the multilinguals. In the monolinguals, the range of proficiency scores becomes less variable in the older age groups. How much cumulative SL exposure does it take for the mean performance of multilinguals to fall within that shrinking monolingual range (after controlling for other aspects of the language experience and for cognitive differences)? We address this question with binary partition models.

### 3.1 Binary Partition Models

The middle column of Table 4 shows, for each measure of SL proficiency, the number of months of cumulative SL exposure beyond which the binary indicator variable was no longer a significant predictor of SL proficiency (with all subsequent cut‐off points tested above that value consistently returning non‐significant results) after controlling for the relevant covariates. A more conservative analysis is shown in the rightmost column of Table 4, excluding children who might at risk for language impairment (as explained in the Data Exclusions section). The NA values will be explained and interpreted in the Discussion section.

**TABLE 4:** Thresholds of cumulative SL exposure beyond which the multilingual–monolingual performance gap was no longer statistically significant, across SL proficiency measures. The full sample includes all children. The restricted sample excludes children at risk for language impairment.

SL proficiency	Cumulative SL exposure threshold full sample	Restricted sample
Identical Repetitions	85	93
Target Repetitions	79	83
Grammatical Attempts	59	65
Vocabulary Breadth	*NA*	*NA*
Vocabulary Depth	91	91

### 3.2 Broken Stick Models

This second set of models sought to determine, for each proficiency measure, the breakpoint from which cumulative SL exposure was no longer correlated with SL proficiency (i.e., the slope became horizontal).

Table 5 reports the breakpoints in the regression line modelling the relationship between cumulative SL exposure and SL proficiency in the multilingual children. The middle column reports the breakpoints from models fitted on all children, and the rightmost column reports those from models excluding the children at risk for language impairment. The interpretability of the breakpoints depends on the slopes on either side. If the confidence interval around a slope estimate crossed zero, that slope was considered horizontal, indicating no discernible relationship between cumulative SL exposure and the relevant SL outcome measure. A meaningful breakpoint requires a non‐horizontal slope before it (reflecting a positive correlation between cumulative SL exposure and proficiency) and a horizontal slope after it (indicating that the correlation is no longer significant). If this pattern is observed, the breakpoint can be interpreted as a developmental threshold.

**TABLE 5:** Breakpoints in the relationship between cumulative SL exposure and SL proficiency outcomes, in the multilinguals. The full sample includes all children. The restricted sample excludes children at risk for language impairment.

SL proficiency	Breakpoints	
	Full sample	Restricted sample
SR.identical	57	56
SR.target	61	58
SR.grammatical	58	58
Voc.breadth	7	46
Voc.depth	62	59

The estimates and confidence intervals for the SR outcomes and for vocabulary depth are shown in Figure 4. Those for vocabulary breadth are shown in Table 6.^9^ The breakpoints for vocabulary depth are non‐interpretable because the slopes are horizontal on both sides of the breakpoint. For vocabulary breadth, the very early breakpoint in the full sample is uninterpretable because the slope preceding it is horizontal (as seen in Figure 4). In the restricted sample, the slope before and after the breakpoint are horizontal. The vocabulary breadth results are therefore uninterpretable, irrespective of whether the full or the restricted sample is considered. This uninterpretability is further reflected in the confidence intervals around the breakpoint locations themselves: for vocabulary depth, the CIs span approximately 55 months for monolinguals and 46 months for bilinguals, indicating that the breakpoint location is poorly identified by the data. For vocabulary breadth in the full sample, the estimated breakpoint of 6.6 months for bilinguals, with an implausibly narrow CI (5.6–7.7 months), is consistent with a model artifact rather than a substantive threshold. By contrast, the breakpoint estimates were highly consistent across the three morphosyntax scoring methods: monolingual thresholds ranged from 71 to 80 months (95% CIs: 56–93 months across models) and bilingual thresholds from 57 to 61 months (95% CIs: 36–80 months across models), providing convergent evidence for the robustness of these estimates.

**FIGURE 4:**
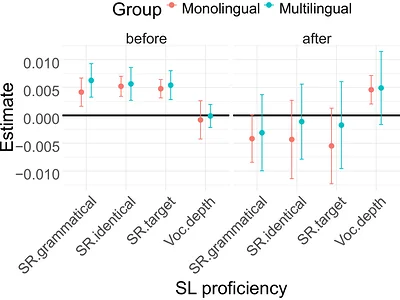
Confidence intervals for the slopes before and after the breakpoint in multilinguals and monolinguals, indicating whether or not they are different from horizontal (i.e., whether or not they cross zero).

**TABLE 6:** Slopes in the broken stick models before and after the breakpoint for vocabulary breadth outcome, showing the 95% confidence intervals.

Group	Slope	Est.	95% CI	
Multilingual	before	−39.361	−456.730	378.010
Multilingual	after	0.246	0.098	0.395
Monolingual	before	−4.120	−49.707	41.466
Monolingual	after	0.680	0.386	0.975

To assess whether multicollinearity between cumulative SL exposure and the richness‐of‐experience predictors could account for the non‐significant slope observed in the broken stick model, we computed generalised variance inflation factors (GVIFs) for the vocabulary breadth model. Scaled GVIFs for all predictors were low (range: 1.03–1.66), with cumulative SL exposure returning a scaled GVIF of 1.17, indicating negligible shared variance with the other predictors (full results in Supplementary Materials). Collinearity can therefore be ruled out as an explanation: the near‐zero coefficient for cumulative SL exposure in the vocabulary breadth model is a reliable estimate.

The non‐significant pre‐breakpoint slopes for vocabulary could be due to a continuity constraint imposed by the broken stick model: the second segment must begin exactly where the first ends. When low‐scoring children are concentrated at the lower end of the exposure range, this pulls the first segment downward, and the second segment must then rise to reach children at higher exposure levels. This is not because genuine developmental growth is occurring, but as a consequence of the model's structural constraint. This is a known property of piecewise regression under uneven predictor distributions.

The apparent contradiction between the binary partition threshold of 91 months for vocabulary depth and the horizontal slopes in the broken stick model is explained by the fact that these two methods address different questions. The binary partition asks whether children above and below a given cut‐off differ significantly as groups; the broken stick asks whether the continuous slope of the exposure‐outcome relationship changes at a particular point. A flat slope across the exposure range in multilinguals is compatible with a persistent group‐level difference relative to monolinguals that only closes at a late threshold. The two results are therefore not logically inconsistent.

## 4 Discussion and Implications

Our first research question was to identify the amount of cumulative SL exposure beyond which multilinguals can, on average, be expected to perform within the range of monolingual peers in terms of SL proficiency. We used a binary variable to partition the children in two groups according to amount of cumulative SL exposure they had experienced (which, for monolinguals, was equivalent to their age). For each proficiency measure in turn, we fitted a series of linear regression models including that binary variable (as our main predictor of interest) alongside the relevant control variables, which had been identified in our previous research. The models were fitted 49 times, using each time a different cut‐off point for the binary variable. We reported the cut‐off point from which the binary partition variable was no longer a significant predictor of SL proficiency.

In terms of morphosyntax, this set of models suggests that a minimum of 59 month‐equivalents of cumulative SL exposure was required for the mean performance of multilingual children to fall within the range of monolingual peers.^10^ This is for the most lenient measure: the one which just requires the production of a grammatical sentence, even if the target structure is not produced or if the child omits or replaces certain words. If we focus on the accurate repetition of the target syntactic structure, the threshold is later, at 79 month‐equivalents of cumulative SL exposure. For the strictest measure of sentence repetition accuracy, which required the verbatim repetition of the prompt and is more taxing in terms of verbal memory, the threshold required was of 85 month‐equivalents. If children who might be at risk for language impairment were excluded (based on their Q‐BEx Risk Factor index), the thresholds were a bit higher: 65 months for grammatical attempts, 83 for target structures, and 93 months for identical repetitions. Note that only four multilinguals in our sample had experienced at least 80 month‐equivalents of cumulative SL exposure (a point we return to below).

In terms of vocabulary breadth, the picture looked quite different. No critical threshold of cumulative SL exposure could be identified within this age range: even at 94 month‐equivalents of cumulative exposure (the maximum for the multilinguals in our sample), there remained a significant difference between monolinguals and multilinguals. The NA values in Table 4 reflect the fact that the indicator variable remained a significant predictor of vocabulary breadth even at the highest levels of cumulative SL exposure. These findings are in line with research showing persistent differences between multilingual and monolingual children in both receptive and expressive vocabulary skills (e.g., Paradis and Jia 2017; Whiteside et al. 2017). In a recent study (Dixon et al. 2022) pooling data from 434 multilingual learners and 342 monolingual peers (UK primary, ages 4;9–11;5) which combined six cross‐sectional and four longitudinal datasets, multilingual pupils showed persistently lower vocabulary scores than monolinguals. There was some convergence in receptive vocabulary by the end of primary school, but expressive vocabulary gaps remained. Convergence in receptive vocabulary only appears in data that span a longer developmental window, underscoring the protracted nature of ‘catching up’ for vocabulary. While research from large population‐based studies investigating the language and academic outcomes for bilingual children is rare, one such study in Australia (Armstrong et al. 2025), which tracked vocabulary and academic outcomes in a cohort of 1801 pupils (126 bilinguals) at ages five and ten, reported that at age 5, bilinguals had lower receptive English vocabulary than monolingual peers. No differences were found between groups in receptive vocabulary at 10 years of age.

For vocabulary depth, the partition analysis identified a threshold of 91 month‐equivalents, unaltered by the exclusion of children at risk for language impairment. However, only two multilingual children in our sample exceed this threshold, which means the result lacks empirical grounding in the available data and should not be interpreted as a reliable estimate. A broader sample including more older multilingual children will be required to investigate this question meaningfully.

A limitation of this first set of analyses is that it focuses on the difference in means between the two groups defined by the binary partition variable, allowing a discontinuity in the relationship between predictor and outcome: the difference in means is not modelled (akin to non‐continuous development). As the youngest monolinguals in our sample have already experienced 60 month‐equivalents of cumulative SL exposure,^11^ the thresholds identified are therefore conservatively high. This is addressed in the second analysis. Our second research question asked from what point cumulative SL experience no longer correlated with SL proficiency. We addressed it using broken stick models, in which the cross‐sectional measurements of cumulative SL exposure are interpreted as a proxy for a developmental effect. Contrary to the binary partition approach above, the broken stick approach treats the horizontal axis as a continuous measurement scale, such that the slope before the breakpoint point is joined up with the slope after the breakpoint: there is no discontinuity, just a change in the relationship between the two variables of interest.

In each model of sentence repetition outcomes, the slope before the breakpoint was significantly different from zero (both for the monolinguals and the multilinguals), as seen by the fact that their confidence interval did not cross the zero line. After the breakpoint, the slope was no longer different from zero, that is it could be considered horizontal. This means that, after the cut‐off point, cumulative SL exposure was no longer a significant predictor of SL morphosyntax outcomes. Furthermore, the confidence intervals for the monolinguals overlapped with those of the multilinguals, indicating that the slopes were parallel for the two groups up to the breakpoint.^12^ Together, the findings support an interpretation in which the relationship between exposure and morphosyntax becomes asymptotic: early increases in cumulative exposure are associated with substantive gains, but beyond the 5–6‐year range of cumulative exposure, multilinguals with equivalent SL experience approach the monolingual developmental trajectory.

Similarly to what was observed in the first set of models, vocabulary measures do not yield conclusive results. The slopes before and after the breakpoints are close to horizontal, which renders the breakpoint meaningless in relation to our research question.

Bringing together the two analyses, the breakpoints in the relationship between cumulative SL exposure and SL morphosyntax occur earlier than suggested by the binary partition models, that is from 57 month‐equivalents of cumulative SL exposure. After that point, there is no longer a significant correlation between cumulative exposure and morphosyntax outcomes (while controlling for relevant factors). Our overall interpretation is that approximately five years of full‐month‐equivalents SL experience (i.e., rounded up from 57 full‐month‐equivalents) constitute a point of diminishing returns for morphosyntax, after which children's performance is no longer strongly constrained by the amount of exposure.

The contrast between the conclusive morphosyntax results and the less tractable vocabulary findings is itself informative: it points to genuine domain‐specificity in the way cumulative SL exposure shapes language development, with morphosyntax appearing more directly and uniformly driven by the quantity of SL input than vocabulary, whose outcomes reflect a more distributed set of influences that the present data (and perhaps any single predictor) cannot fully capture. Indeed, part of the explanation for the inconclusive results regarding SL vocabulary outcomes is the great importance of other predictors in the relevant models: Cognitive factors and the richness of the language experience account for most of the variability in vocabulary outcomes in these models, leaving little room to detect the impact of differences in cumulative SL exposure. Within the age and exposure range captured here, cumulative SL exposure is not the primary driver of individual differences in lexical breadth. By contrast, vocabulary depth (measured through the Word Classes test) did show a model‐identified threshold (91 months), but only two multilingual children had exposure levels above this point, precluding firm conclusions. In a study that investigated the role of cognitive and environmental predictors of vocabulary outcomes in the same group of multilingual children Serratrice et al. (2026), verbal working memory and verbal short‐term memory consistently emerged as the most significant predictors of both vocabulary breadth and vocabulary depth. While current and cumulative exposure to the SL were among the predictors, what had a larger protective impact on vocabulary outcomes was the richness of the children's experience in the SL (see also Unsworth et al. 2025); for instance, out‐of‐school instruction and participation in organised societal‐language activities were found to boost vocabulary size among children with weaker verbal short‐term memory. That multilinguals in this study attended monolingual educational systems also means their vocabulary development is anchored in school‐based exposure, which may homogenise growth trajectories irrespective of earlier variation in cumulative exposure. The lack of identifiable thresholds thus does not imply that exposure is irrelevant; rather, it indicates that vocabulary outcomes reflect a more distributed set of influences that dilute the unique contribution of cumulative SL experience.

What can education and health professionals conclude from our findings, when assessing the language support needs of multilingual children educated monolingually in the SL? We start with an important caveat: The thresholds identified in this study were derived from group−level comparisons and therefore describe population‐level tendencies, not individual trajectories. They should inform, rather than determine, clinical judgment about any individual child. The thresholds have to be interpreted within the context of other factors affecting proficiency outcomes in that language (as reviewed in the Introduction). As we controlled for these factors in our analyses, the thresholds need to be interpreted as valid for children characterised as ‘average’ on all these other factors. For example, a child who has experienced low levels of Richness in their language environment is likely to need more cumulative exposure than suggested by the threshold. Bearing this caveat in mind our advice is as follows.

Before multilingual children have experienced at least 57 month‐equivalents of cumulative SL exposure, more difficulties with SL morphosyntax are naturally expected compared with monolingual children with equivalent cumulative SL exposure. By contrast, if a multilingual child exhibits poor morphosyntactic skills despite substantial cumulative SL exposure (tentatively, beyond approximately 93 month‐equivalents of SL exposure), referral for specialist assessment should be considered, particularly where other indicators of language difficulty are also present. Earlier identification remains preferable where possible; comparison with multilingual peers at similar levels of cumulative SL exposure, alongside other indicators such as early language development milestones and parental concern, can help prompt timely referral (see, e.g., De Cat, Tuller, et al. 2026).

As a global recommendation, our results suggest that multilingual children will benefit from support with the school language until they have experienced at least 93 month‐equivalents of cumulative SL exposure, and possibly throughout primary school. This is in line with the large cohort study by Zhang and Winke (2025), who found that child English Learners in the US need between 2 and 8 years to reach the English proficiency threshold set as target in their state's education authority. Similarly, in England, a recent report on the educational attainment of pupils with English as an Additional Language (Lindorff et al. 2025) made policy recommendations for continued language support to multilingual learners to attain the level of English language proficiency that is predictive of their academic success (Hessel and Strand 2023).

The domain‐specificity observed here underscores the importance of assessing multiple linguistic subsystems rather than relying on a single global indicator of proficiency. Targeted approaches that focus on developing vocabulary breadth and depth—not just for multilingual pupils but for monolinguals alike—are likely an area to prioritise to boost oral language skills and literacy skills that underpin academic success and improve life chances.

A practical question concerns how the thresholds reported here can be implemented by speech and language therapists and educators. Calculating cumulative SL exposure and interpreting it in the light of other relevant individual factors may seem demanding, but this is directly addressed by the Q‐BEx questionnaire (De Cat et al. 2025), which is freely available (https://q‐bex.org) and designed to be completed by parents. Q‐BEx not only estimates cumulative SL exposure in month‐equivalents (weighting periods of exposure by the proportion allocated to each language, so that intensity and proportion of SL and heritage language exposure are explicitly captured) but generates individualised, evidence‐informed reports contextualising the child's exposure profile in light of other relevant factors, including richness of language experience and a Risk Factor index for language impairment. The guidance provided at the end of each Q‐BEx report is specifically designed to support the kind of contextualised interpretation the present findings call for, translating research benchmarks into accessible clinical guidance at the level of the individual child. The thresholds reported here and the benchmarks embedded in Q‐BEx reports are in this sense mutually reinforcing: the present findings provide the empirical grounding for those benchmarks, while Q‐BEx provides the practical delivery mechanism that makes them actionable in clinical and educational settings. A sample report can be downloaded from https://www.q‐bex.org/slts/resources/.

Turning now to the limitations of our study, we would like to highlight three points. Firstly, the measures targeted structural properties of morphosyntax and vocabulary rather than academic language or discourse‐pragmatic competencies. These latter domains—crucial for school success—likely require substantially more extensive experience to mature and may show different exposure–outcome dynamics (e.g., Zhang and Winke 2025).

Secondly, some of the control variables included in our predictive models might in fact be mediators of the effect of cumulative SL exposure on SL outcomes, that is variables through which the effect of cumulative SL exposure is transmitted (De Cat and Unsworth 2023). For example, the current frequency of literacy activities in the SL is likely to be correlated with the frequency of such activities earlier in the child's life. Uncovering the causal effect of cumulative SL exposure on SL outcomes would require taking such mediator variables into account (as they contribute to the total effect of cumulative SL exposure on SL outcomes). This is however beyond the scope of this paper, and should ideally be investigated based on longitudinal data.

Finally, our study focused on children between 5 and 9 years of age, the onset of formal education. Data from older children will be required to determine with certainty the point at which the gap is closed. Our results suggests that it takes at least 93 month‐equivalents of cumulative SL exposure to fully reach that level of morphosyntactic competence. Longer‐term follow‐up, ideally extending to early adolescence, will be necessary to map the full developmental trajectory and determine when the asymptote is reached. A related limitation concerns the difficulty of disentangling cumulative SL exposure effects from developmental maturation in a cross‐sectional design. In monolingual children, cumulative SL exposure and age are perfectly correlated by definition, meaning the two cannot be separated within the present data. In multilingual children the correlation is imperfect, which is why cumulative exposure is a more informative predictor than chronological age for this group, but a developmental component necessarily remains. The cognitive control variables included in our models (working memory, non‐verbal IQ) partially index developmental maturity independently of language exposure, and the parallel slopes observed for monolinguals and multilinguals up to the breakpoint are consistent with a genuine exposure effect. Nevertheless, the thresholds identified should be understood as reflecting the combined influence of cumulative SL exposure and developmental maturation. Longitudinal data tracking the same children over time would be needed to disentangle these effects more cleanly.

In spite of these limitations, our study is innovative in several aspects. First, it takes variability within monolinguals into account and avoids the use of arbitrarily determined cut‐offs to determine monolingual‐like performance. Second, it includes a range of environmental and cognitive factors to control for their impact on language outcomes in the statistical models, not only in multilinguals but also in monolinguals. This makes it possible to consider a diverse cohort in terms of cognitive and socio‐economic profiles. That diversity is also manifested in the rich cross‐linguistic profile of our cohort (spanning three SLs and featuring a total of 38 different heritage languages), their age range (5 to 9 year of age), their age of onset of SL exposure (including both simultaneous and sequential bilinguals), and a very wide spectrum of SL exposure. Third, we considered a range of proficiency measures, spanning different aspects of morphosyntax and receptive vocabulary. Finally, this study is innovative in its modelling methods, combining recursive binary partition models and broken stick models to shed light on the narrowing gap between monolingual‐like and multilingual‐like performance, and to identify when, in terms of cumulative SL exposure, multilingual children can be expected to have caught up with their monolingual peers.

## Funding

This project was supported by a grant from the Economic and Social Research Council (ES/S010998/1).

## Ethics Statement

The study was approved by the ethics committee of each participating university: Tours (Comite d’éthique de la recherche Tours‐Poitiers: 2021‐09‐04), Leeds (the Faculty of Arts’ Humanities and Cultures Research Ethics Committee: 21‐006 Amd2), and Nijmegen (Ethics Assessment Committee of the Faculty of Arts and the Faculty of Philosophy, Theology and Religious Studies: 2021–9263). Written informed consent was obtained from the parents of each child participant.

## Conflicts of Interest

The authors have nothing to report.

## Supporting information




**Supporting Information**: jlcd70330‐sup‐0001‐SuppMat.html

## Data Availability

The data and R scripts that support the findings of this study are available via the OSF at http://osf.io/4×3dq/overview?view_only=cea5212625f5461ead3b2c3c02384786

## Endnotes

1 Language input is one among many factors that affect language development in multilingual learners (see e.g., Paradis 2023 for a recent review). In this paper, we focus on the quantity of language input and control for other factors, as will be explained in the methods section.

2 The breakdown by country is as follows. UK: mean = 84, sd = 12; NL: mean = 82, sd = 12; FR: mean = 81, sd = 13. See the Supplementary Materials for distribution plots by country and group (monolinguals vs. multilinguals).

3 The heritage languages represented are: Afrikaans, Albanian, Arabic, Bangla, Bulgarian, Chinese, Creole, Czech, Dutch, English, Filipino, French, Frisian, German, Greek, Gujarati, Hindi, Hindko, Italian, Japanese, Kurdish, Latvian/Lithuanian, Lingala, Manouche, Marathi, Mirpuri, Papiamento, Polish, Portuguese, Punjabi, Romanian, Russian, Spanish, Telugu, Turkish, Ukrainian, Urdu, Zulu. Note: Latvian/Lithuanian is how the parent named their child's heritage language.

4 For a demonstration of how cumulative exposure is calculated by the Q‐BEx questionnaire, see resources on https://www.q‐bex.org/, https://www.q‐bex.org/researchers/using‐the‐questionnaire/.

5 In the validation study for the Risk Factor (De Cat et al. 2026), a cut‐off of 3 achieved a specificity of 80% in the identification of language impairment.

6 Assuming 6 h of school per day (out of a total of 14 h awake), 5 days per week for 39 weeks per year, and 100% SL exposure at school, any child in monolingual SL education should have a minimum of approximately 23% SL exposure (on average over the year). We therefore consider a putative SL proportion below 23% to be unrealistic.

7 See Prévost et al. (2026) about the different number of items across language versions.

8 Children at risk for language impairment are excluded from this figure. As there are only seven 9‐year‐olds, we group them together with the 8‐year‐olds in the figure. This doesn't affect the analyses in any way.

9 The vocabulary breadth scores were not expressed as a percentage, and their magnitude would have rendered the other measures illegible if included in the same figure.

10 This is the earliest breakpoint reported in Table 4 for the full sample.

11 We recruited children from the onset of formal education, considering that this coincides with a boost in SL exposure. Before that point, we assumed that the variability among monolinguals would be too high.

12 Separate breakpoints can technically be estimated for monolinguals, but their interpretation is not analogous to the multilingual one: in monolingual children, cumulative SL exposure and chronological age are perfectly correlated by definition, so a monolingual breakpoint cannot be attributed to exposure per se and would be indistinguishable from a maturational threshold. It is precisely because cumulative SL exposure varies independently of age in multilinguals that the breakpoint carries its interpretive value as an exposure threshold.
