# Supplementary material for: A Data‐Driven Approach to the Issue of ‘Catching Up With Monolinguals’
Source: Int J Lang Commun Disord. 2026 Sep 29;61(6):e70330. doi: 10.1111/1460-6984.70330 (PMC13621806; doi:10.1111/1460-6984.70330)
Supplement: Supplementary file 1 [file JLCD-61-0-s001.html]

Supplementary materials


# Supplementary materials

- Age distribution by
  country and by group
- Distribution of
  outcome measures by country
  - By-country and
    by-group outcome measures
  - Relationship
    between cumulative SL exposure and outcomes, by country, in the
    multilinguals
- Control variables
  included in the models
  - Sentence
    repetition
    - Identical repetitions
    - Target
      repetitions
    - Grammatical attempts
  - Vocabulary
    - Vocabulary
      breadth
    - Vocabulary
      depth
- Multicollinearity
  diagnostics (in relation to vocabulary breadth)
- References

# Age distribution by country and by group

# Distribution of outcome measures by country

## By-country and by-group outcome measures

The plots below show the distribution of all the outcome measures by
group (monolinguals vs multilinguals) and by societal language (as a
proxy for country of residence). By-country differences are captured in
our analyses by including Societal Language as a control variable.

## Relationship between cumulative SL exposure and outcomes, by country, in the multilinguals

The by-country scatterplots show that the positive relationship
between cumulative SL exposure and morphosyntactic accuracy is
consistent in direction across all three countries. The French sample
shows a steeper apparent slope in some of the plots, but given the
compressed exposure range and small sample size (n=19) in that country,
this should be interpreted with caution: the wide confidence interval
reflects considerable uncertainty around that estimate. There is no
evidence from these plots that the exposure-outcome relationship
operates qualitatively differently across countries, which supports the
validity of the pooled analysis.

# Control variables included in the models

- SL: societal language (English, Dutch, or French).
- Non-verbal IQ: The Matrix Reasoning sub-test from the Wechsler
  Intelligence Scale for Children (WISC-IV) (Wechsler, 2003) or from the
  Wechsler Preschool Primary Scale of Intelligence (WPPSI) (Wechsler,
  2012) – depending on the child’s age – was administered as a proxy
  measure for non-verbal intelligence and analytical reasoning. Children
  are shown coloured matrices or visual patterns with something missing,
  and they are asked to choose the missing piece from several available
  options. The test is untimed and we used scaled scores in the analyses.
  **NVI.bin** was a dummy variable identifying children who
  performed below the normal range on the non-verbal intelligence test.
  The cutoff point was a scaled score below 4 on the relevant WISC or
  WPPSI measure.
- Verbal short term memory and working memory – digit recall tasks:
  Verbal short term and verbal working memory were assessed using a digit
  recall task developed by Hill et al. (2021). The span range for the
  forward digit recall (FDR) is 3-6 items, for the backward digit recall
  it is 2-5 items; each span length includes 4 trials for a total of 16
  trials in the FDR and 16 trials in the BDR. There is no stopping rule,
  and skipped trials are counted as missing data. The scoring includes an
  accuracy score per span; percent correct and an average score across
  spans. In the current study we used a percentage correct score which
  reflected any correctly repeated number in the correct
  position.
- Phonological skills – non-word repetition tasks: We used the
  available adaptations of the LITMUS Quasi Universal Non-Word (QU-NWR)
  repetition task for French, Dutch, and British English from the Language
  Impairment Testing in Multilingual Settings tool kit (LITMUS, 2024).
  Each QU-NWR task has a set of language-independent items that are
  plausible in most languages of the world (N = 13 for French; N = 24 for
  Dutch; N = 13 for English) and a set of language-dependent items that
  include elements of phonological complexity that are language-specific
  (N = 18 for French; N = 32 for Dutch; N = 18 for English). All items are
  composed of three vowels ([i], [a] and [u]) and five consonants (a
  labial stop, a dorsal stop, a labial fricative, a liquid, and a coronal
  fricative). Syllable structure includes CV, CCV, and CVC. Scoring is
  binary (0=incorrect/1 = correct) and items are scored at the word level
  rather than at the phoneme level. Percentage correct scores are used in
  the analyses.
- Vocabulary breadth: In English we assessed children’s vocabulary
  size using the British Picture Vocabulary Scale-Third Edition - BPVS –3,
  (Dunn et al., 2009), a picture matching task measuring single word
  comprehension. In Dutch and in French we used adaptations of the Peabody
  Picture Vocabulary Test: the Peabody Picture Vocabulary Test-III-NL
  (PPVT-III NL; Schlichting, 2005) for Dutch, and the Échelle de
  Vocabulaire en Images Peabody (ÉVIP) (Dunn et al., 1993) originally
  adapted to French in Canada. Raw scores were used in the
  analyses.
- Vocabulary depth: Children’s depth of word knowledge was assessed
  in English with the Word Classes sub-test from the Clinical Evaluation
  of Language Fundamentals 5th Edition UK (Wiig et al., 2017) and with the
  equivalent Word Classes sub-test in the Clinical Evaluation of Language
  Fundaments, 4th edition–NL (CELF4-NL) (Kort et al., 2008) in Dutch, and
  CELF 4—Batterie d’évaluation des fonctions langagières et de
  communication (Adaptation française ECPA) in French (Wiig et al., 2019).
  In this task children are initially presented with a set of 3 pictures
  and then 3 or 4 words, and are asked to select the two that go together
  according to semantic class (e.g. cat, cow, kitten), location
  (e.g. books, porch, library, cave), composition (e.g. rock, shirt, pie,
  cloth), synonyms (e.g. silent, quiet, gentle, tired), object function
  (e.g. key, razor, hammer, nail), or opposite (e.g. smooth, wise, rough,
  heavy). The task includes 40 items. Percentage correct scores out of 40
  were used in the analyses.
- Onset of word production: indicator of whether the onset of word
  production was very delayed (later than 25 months of age), delayed
  (between 16 and 24 months of age), or within the typical range.
- Onset of word combinations: indicator of whether the onset of
  productive syntax was very delayed (later than 31 months of age),
  delayed (between 25 and 30 months of age), or within the typical
  range.
- Early language concerns: binary indicator of whether the parents
  or caregivers were concerned about the child’s language development
  before the age of 4.
- Language entropy: Using the languageEntropy R package (Gullifer
  & Titone, 2018), we derived a measure of language entropy: an
  estimate of uncertainty regarding the language choice expectations in
  each context (interpreted as proxy for language mixing). This variable
  was scaled.
- **Older siblings** was the number of older siblings,
  distributed as shown in the Table below.
- Parental education was 5-level ordinal variable determining the
  highest level of formal education achieved by one of the caregivers in
  the households. The ordered levels were: none, primary, secondary,
  post-secondary, and university. The variable was transformed into a
  numeric variable (ranging from 0 to 4) for ease of interpretability of
  the model summaries.

```
##               
##                 0  1  2  3  4
##   Monolingual   0  2 12 12 69
##   Multilingual  1  5 17 20 80
```

- Seven aspects of the richness of the child’s language experience
  were considered. This consisted in frequency data, originally on a
  5-point Likert scale with the following levels: (almost) never, once or
  twice a month, once or twice a week, several times a week and every day.
  The variables were transformed into numeric ones (ranging from 0 to 4).
  The variables included the frequency of literacy-related activities in
  the SL (writing, reading, in- and out-of-school lessons about the SL,
  homework in the SL), social activities in the SL (organised activities,
  time with friends), and the approximate number and proficiency of
  interlocutors in the SL.

|  | Mean | SD |
| --- | --- | --- |
| Forward.digit.recall | 0.68 | 0.15 |
| Backward.digit.recall | 0.56 | 0.18 |
| Non.word.repetition | 0.70 | 0.22 |

Below, we list for each outcome measure the control variables
included in the regression models. This was informed by previous
analyses of the same dataset, in which we identified the optimal
predictive model for each outcome measure (see references in the
article).

## Sentence repetition

### Identical repetitions

SL, Forward digit recall, Older siblings, Writing frequency in the
SL, Tech activities in the SL, Organised activities in the SL, Reading
frequency in the SL, Out-of-school lessons about the SL

### Target repetitions

SL, Forward digit recall, Backward digit recall, Phonological skills,
Older siblings,  
Time with friends in the SL, Organised activities in the SL,
Out-of-school lessons about the SL, In-school lessons about the
SL,  
Highest parental education in the household

### Grammatical attempts

Forward digit recall, Backward digit recall, NVI.bin, Phonological
skills, Older siblings, Onset of word production, Language entropy,
Organised activities in the SL, Tech activities in the SL, Writing
frequency in the SL, Highest parental education in the household

## Vocabulary

### Vocabulary breadth

SL, Forward digit recall, NVI.bin, Phonological skills, Older
siblings, Writing frequency in the SL, In-school lessons about the SL,
Out-of-school lessons about the SL, Organised activities in the SL,
Highest parental education in the household

### Vocabulary depth

SL, Forward digit recall, Backward digit recall, NVI.bin, Older
siblings, Onset of word production, Onset.syntax.RF,
Language.concerns.before.age.4, Phonological skills, Reading frequency
in the SL, Writing frequency in the SL, Homework.freq.SL, In-school
lessons about the SL, Out-of-school lessons about the SL, Tech
activities in the SL, Time with friends in the SL, Organised activities
in the SL, Highest parental education in the household

# Multicollinearity diagnostics (in relation to vocabulary breadth)

Multicollinearity among predictors was assessed using the Generalized
Variance Inflation Factor (GVIF; Fox & Monette, 1992), implemented
via the car package in R (Fox & Weisberg, 2019). GVIF is used in
place of standard VIF to account for categorical predictors with more
than one degree of freedom (here, SL with Df = 2). To allow comparison
across predictors regardless of degrees of freedom, scaled GVIF values
[GVIF^(1/(2·Df))] are reported; these are interpretable on the same
scale as the square root of standard VIF.

All scaled GVIF values fell below 1.70 (range: 1.03–1.66), indicating
no problematic multicollinearity. No predictor approached the
conventional thresholds of concern (scaled GVIF > 2.24, equivalent to
VIF > 5).

Variance Inflation Factors, showing the Generalized Variance Inflation
Factor, Degrees of Freedom, and GVIF^(1/(2·Df))

|  | GVIF | Df | Scaled GVIF |
| --- | --- | --- | --- |
| SL.cumulative.exposure.mo | 1.376 | 1 | 1.173 |
| FDR\_overall | 1.804 | 1 | 1.343 |
| NVI.bin | 1.145 | 1 | 1.070 |
| NWR | 2.766 | 1 | 1.663 |
| Highest.parental.educ.n | 1.178 | 1 | 1.085 |
| SL | 3.451 | 2 | 1.363 |
| Older.siblings | 1.057 | 1 | 1.028 |
| Writing.freq.SL | 1.446 | 1 | 1.202 |
| In.school.lessons.about.SL.freq | 1.127 | 1 | 1.062 |
| Out.school.lessons.about.SL.freq | 1.216 | 1 | 1.103 |
| Org.activities.freq.SL | 1.229 | 1 | 1.108 |

# References

Fox, J., & Monette, G. (1992). Generalized collinearity
diagnostics. *Journal of the American Statistical Association*,
*87*(417), 178-183. https://www.jstor.org/stable/2290467

Fox, J., & Weisberg, S. (2019). An R companion to applied
regression (3rd ed.). Sage. https://www.john-fox.ca/Companion/
